# *Taenia lynciscapreoli* in Eurasian Lynx: New Taeniid Record for Romania

**DOI:** 10.3390/pathogens15050468

**Published:** 2026-04-25

**Authors:** Maria Monica Florina Moraru, Ana-Maria Marin, Dan-Cornel Popovici, Azzurra Santoro, Federica Santolamazza, Radu Blaga, Kalman Imre, Narcisa Mederle

**Affiliations:** 1 Department of Parasitology and Parasitic Diseases, Faculty of Veterinary Medicine, University of Life Sciences “King Mihai I”, 300645 Timisoara, Romania; mariamoraru@usvt.ro (M.M.F.M.); narcisamederle@usvt.ro (N.M.); 2 Forestry Faculty, Transilvania University Brasov, 500123 Brasov, Romania; danpopovici30@yahoo.com; 3 WHO Collaborating Centre for the Epidemiology, Detection and Control of Cystic and Alveolar Echinococcosis (One Health), Istituto Superiore di Sanità, 00161 Rome, Italy; azzurra.santoro@iss.it (A.S.); federica.santolamazza@iss.it (F.S.); 4 European Union Reference Laboratory for Parasites (EURL-P), Foodborne and Neglected Parasites Unit, Department of Infectious Diseases, Istituto Superiore di Sanità, 00161 Rome, Italy; 5 Anses, INRAE, Ecole Nationale Vétérinaire d’Alfort, Laboratoire de Santé Animale, BIPAR, F-94700 Maisons-Alfort, France; radu.blaga@vet-alfort.fr; 6 Department of Animal Production and Veterinary Public Health, Faculty of Veterinary Medicine, University of Life Sciences “King Mihai I”, 300645 Timisoara, Romania; kalmanimre@usvt.ro

**Keywords:** *Taenia lynciscapreoli*, Eurasian lynx, Romania

## Abstract

The Eurasian lynx (*Lynx lynx*) is an apex predator and an important sentinel for trophically transmitted helminths acquired via predation on wild ungulates. On 2 March 2022, an adult male lynx that was road-killed in the Apuseni Mountains (Surducel hunting ground, Bihor County) was collected, frozen for biosafety, and a necropsy was performed. Taeniid cestodes were detected, with a total intestinal burden of nine adult specimens. Genetic analyses confirmed *Taenia lynciscapreoli*, and the obtained sequences were deposited in GenBank (PV843597, PV855065, PV844409). Phylogenetic inference based on *cox1* assigned the Romanian isolate within the European cluster, distinct from the Chinese isolate, while showing genetic proximity to *Taenia* sp. (MW846305) that have been reported from a lynx in China. This study represents the first molecular identification of *T. lynciscapreoli* in the Eurasian lynx in Romania and, to our knowledge, the first record from Southeastern Europe.

## 1. Introduction

The Eurasian lynx (*Lynx lynx*) sits at the top of the food chain in the European and Asian ecosystems where it lives. Its primary prey consists of wild ungulates (roe deer (*Capreolus capreolus*), red deer (*Cervus elaphus*) and wild boar (*Sus scrofa*)), although it may also consume certain rodent species [1,2,3]. As a large carnivore, the Eurasian lynx can serve as a host for parasite assemblages acquired from its prey, which occupy lower trophic levels. This is particularly relevant for assessing interspecific ecological relationships and trophic transmission, as well as predator–prey dynamics and interactions between intermediate and definitive host. Such information is also valuable for understanding parasite circulation in wildlife populations and its potential relevance to host health [4]. At present, the Eurasian lynx distribution range is largely continuous in Northern Europe (Scandinavia and Russia) but fragmented into small populations in Central and Western Europe [5]. Population densities reported in the Transcarpathian habitats are among the highest across Eurasia, underlining the relevance of the Romanian Carpathian population for the conservation of the species. However, as in other parts of its range, the Eurasian lynx may be exposed to pressures such as habitat degradation, habitat fragmentation, human disturbance, and road mortality. Therefore, the species is strictly protected under Annex IV of Directive 92/43/EEC and Romanian Law 407/2006 on wildlife protection [6]. Although the Eurasian lynx is currently classified as Least Concern on the IUCN Red List, the population inhabiting the Romanian Carpathians is considered one of the most important in Europe in terms of population size and conservation relevance [7]. However, studies on the helminth fauna of this species in Romania remain scarce. Available studies from Romania have focused primarily on parasitic zoonoses in wild carnivores or on cardiopulmonary parasites in wild felids, rather than specifically on cestode diversity in Eurasian lynx. These parasite groups are relevant to host health; however, species-level identification of cestodes using molecular approaches has remained lacking [8,9,10].

*Taenia lynciscapreoli* was first suggested as a distinct taxon in Finland by Lavikainen et al. (2013) [11] and was subsequently confirmed there in 2016 by Haukisalmi et al. (2016) [12] on the basis of morphological and molecular criteria. Given that interspecific morphological differences within the genus *Taenia* may be subtle and therefore prone to misinterpretation, molecular tools such as *cox1* and *nad1* based sequencing are particularly valuable for accurate species identification [13,14].

*Taenia lynciscapreoli* has a heteroxenous life cycle in which Eurasian lynx acts as the definitive host and ungulates—primarily the roe deer—serve as natural intermediate hosts [12,13,14]. New intermediate hosts for *T. lynciscapreoli* have been reported in Sweden, where molecular confirmation of the metacestode in semi-domesticated reindeer (*Rangifer tarandus tarandus*) suggests either accidental infection or a broader ecological plasticity than previously assumed for this parasite [15].

Within the broader aim of improving current knowledge of parasite diversity, host–parasite relationships, and wildlife health in the Eurasian lynx as a protected species in the Carpathian region, we report the first molecular identification of *T. lynciscapreoli* in Romania.

## 2. Materials and Methods

**Figure 1:**
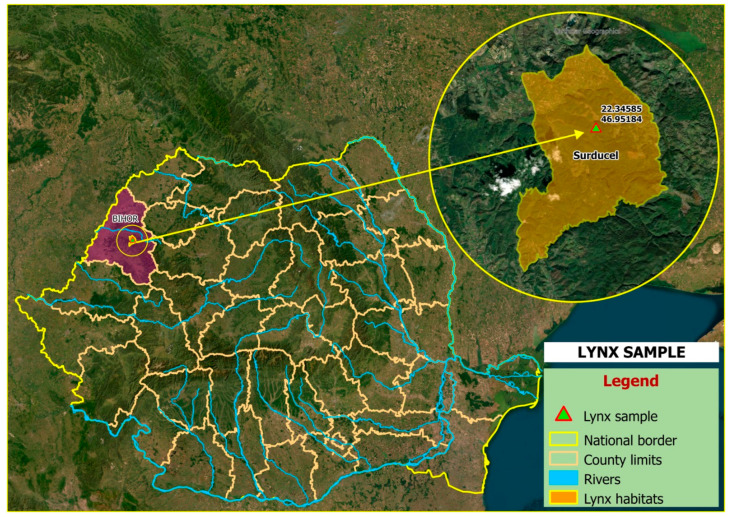
Map showing the location of the sampled Eurasian lynx specimen.

On 2 March 2022, an adult male Eurasian lynx was found dead following a road traffic collision in the Apuseni Mountains (Surducel hunting ground), Bihor County, Romania (46.820240° N, 21.669641° E) (Figure 1). The carcass was transported to the Clinic of Parasitic Diseases, Faculty of Veterinary Medicine, University of Life Sciences “King Mihai I” from Timișoara, for parasitological investigations. Previous to the examination, biosafety procedures were implemented in accordance with international guidance from the World Organisation for Animal Health (WOAH) [16] and the European Food Safety Authority (EFSA) [17], and the carcass was stored at −80 °C for 48 h to inactivate potential zoonotic agents.

The entire small intestine of the animal was removed and placed separately in a 20 L plastic container, then dissected and systematically examined for the presence of adult cestodes (tapeworms). No parasite-associated gross intestinal lesions were observed at necropsy. When parasites were detected, they were carefully recovered using fine forceps, rinsed several times in physiological saline, and kept in 70% ethanol for subsequent morphological assessment and molecular characterization. However, deep freezing affected the structural integrity of the helminths, and standard morphological identification could not be performed [18]. Therefore, the cestode specimens were stored in 70% ethanol for further molecular identification analyses.

Genomic DNA was extracted from proglottids, using the DNeasy Blood & Tissue Kit (Qiagen, Valencia, CA, USA) following the manufacturer’s instructions. A negative extraction control (nuclease-free water) was included in each extraction batch to monitor potential contamination. DNA was stored at −20 °C until further processing. The oligonucleotide primers used for amplification of the mitochondrial gene fragments analyzed in this study are summarized in Table 1.

A fragment of the mitochondrial cytochrome c oxidase subunit 1 (*cox1*) gene was amplified using primers EgCOI 1 and EgCOI 2, as described by Bowles et al. (1992) [19] and modified by Bart et al. (2006) [20]. PCR was performed in a final volume of 30 μL containing 2 μL of template DNA, 15 μL of HotStart PCR Master Mix (Qiagen GmbH, Hilden, Germany), 0.5 μM of each primer, and nuclease-free water to volume (10 μL). The cycling conditions were 95 °C for 15 min, followed by 38 cycles at 94 °C for 30 s, 55 °C for 30 s, and 72 °C for 30 s, with a final extension at 72 °C for 5 min.

A fragment of the mitochondrial *12S rRNA* gene was amplified using primers P60 and P375 according to Dinkel et al. (2004) [21]. PCR was carried out in a total volume of 50 μL containing 5 μL of DNA template, 25 μL of HotStart PCR Master Mix (Qiagen GmbH, Hilden, Germany), 0.5 μM of each primer, and 15 μL of nuclease-free water. Cycling conditions were 95 °C for 15 min, followed by 40 cycles at 93 °C for 60 s, 55 °C for 90 s, and 72 °C for 2 min, and a final extension at 72 °C for 5 min. A no-template control (nuclease-free water) was included in each PCR run.

In addition, a fragment of the mitochondrial NADH dehydrogenase subunit 1 (*nad1*) gene was amplified using primers JB11 and JB12 [22]. Reactions (30 μL) contained 2 μL of DNA, 15 μL of HotStart PCR Master Mix (Qiagen GmbH, Hilden, Germany), 0.5 μM of each primer, and 10 μL of nuclease-free water. The thermal profile consisted of 95 °C for 15 min, followed by 35 cycles at 94 °C for 30 s, 53 °C for 1 min, and 72 °C for 30 s, with a final extension at 72 °C for 5 min.

PCR products were visualized by capillary gel electrophoresis using the QIAxcel system (Qiagen GmbH, Hilden, Germany). Amplicons were purified and Sanger sequenced by GENEWIZ (Leipzig, Germany). Sequence identity was assessed using BLAST ( 2.17.0) against the NCBI database [23]. Sequences were deposited in GenBank under accession numbers PV843597 (*cox1*), PV844409 (*12S rRNA*), and PV855065 (*nad1*). Phylogenetic analysis was reconstructed with maximum likelihood (ML) inference based on an alignment of *cox1* gene sequences, including the sequences obtained in this study as well as sequences available for representative Cestoda species in GenBank. The Jukes-Cantor substitution model was chosen according to the model selection function of the CLC Main Workbench 22.0.2 software (Qiagen) [24]. 1000 bootstrap replicates were performed to estimate the branch robustness.

## 3. Results

Following the necropsy of the Eurasian lynx from the Surducel hunting ground, Bihor County, nine adult cestodes, consistent with the family Taeniidae were detected.

PCR assays, targeting *cox1*, *12S rRNA*, and *nad1*, on the genomic DNA of proglottids yielded positive amplicons. Sanger sequencing of the resulting *cox1*, *12S rRNA*, and *nad1* fragments confirmed species identity as *Taenia lynciscapreoli*.

**Figure 2:**
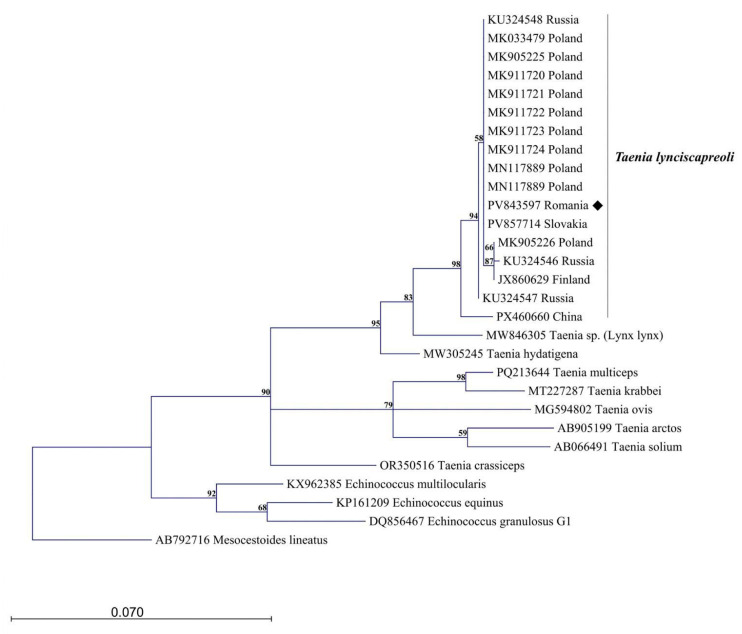
Phylogenetic relationships based on the *cox1* (391 bp) sequence alignments of *Taenia lynciscapreoli* obtained in this study (black diamond), along with selected reference sequences from GenBank representing *T. lynciscapreoli* and other related cestodes. The phylogenetic tree was constructed using the Maximum Likelihood method in CLC Genomics Workbench, displaying only branches supported by bootstrap values > 50% (1000 replicates). Reference sequences are annotated with GenBank accession numbers, scientific names, and country of origin for *T. lynciscapreoli*. The scale bar indicates the number of nucleotide substitutions per site.

The phylogenetic analysis was based on a 391 bp *cox1* sequence. The *cox1* sequence obtained in this study (PV843597) showed 100% identity with the *T. lynciscapreoli* reference sequence MK033479 in GenBank, confirming that the Romanian isolate belongs to this species. In addition, the *nad1* sequence (PV855065) showed 99.39% identity with the *T. lynciscapreoli* reference sequence JX860630. No comparative *12S rRNA* sequence of *T. lynciscapreoli* was available in GenBank, except for the sequence generated and deposited in the present study, which represents the first *12S* record available for this species. In the phylogenetic tree, the Romanian isolate clustered within the European lineage, which is distinct from the Chinese *T. lynciscapreoli* clade. Within the European cluster, a subcluster comprising isolates from Finland, Russia, and Poland was identified; however, the Romanian isolate did not group within this subcluster. The phylogenetic tree also underscores the close relationship between *T. lynciscapreoli* and a novel taeniid species recently detected in a lynx in China, currently referred to as *Taenia* spp. (MW846305) (Figure 2).

## 4. Discussion

The present study represents the first report and molecular characterization of *T. lynciscapreoli* in Eurasian lynx from Romania. Although the necropsy examination involved a single host individual, molecular identification was based on sequencing of a single cestode specimen selected from the nine adult worms recovered at necropsy, using the mitochondrial *cox1*, *12S rRNA*, and *nad1* genes. This finding provides an important contribution to current knowledge of the geographic distribution of this cestode in Europe.

Coprological surveys have reported the presence of *Taenia* spp. in Eurasian lynx from Poland [25] and Finland [26], as well as in the Iberian lynx (*Lynx pardinus*) [27], while necropsy-based investigations have revealed *Taenia* spp. in lynx from Estonia [28].

The Eurasian lynx has been reported as the only definitive host for *T. laticollis* and for an unidentified *Taenia* species in Finland [11] and China [29]. In addition, the bobcat (*Lynx rufus*) is the definitive host for *Taenia rileyi* in Arkansas [30].

The specific prey–predator relationship underlying the transmission of T. lynciscapreoli is supported by the demonstrated conspecificity between adult worms recovered from Eurasian lynx and metacestodes identified in roe deer, as shown morphologically and through *cox1* and *nad1* sequencing in Finland and Poland [12,14].

The use of three genetic markers (*cox1*, *12S rRNA*, and *nad1*) in the present study provides robust certainty for molecular diagnosis. The confirmation of *T. lynciscapreoli* in a Eurasian lynx specimen originating from Bihor County is ecologically plausible, as this area is characterized by extensive forest habitats and substantial densities of roe deer [2], the natural intermediate host for this parasite. Moreover, the ecological similarity between the Western Carpathians and the mountainous regions of Poland—where *T. lynciscapreoli* is well documented—supports the hypothesis of a largely continuous distribution across the European range of the Eurasian lynx [14,31]. Reports from Finland and Russia have shown that this species is widely distributed throughout the natural range of the Eurasian lynx and was likely underdiagnosed previously due to its morphological similarity to other *Taenia* species [12].

Evidence for broader ecological plasticity is provided by Kautto et al. (2022) [15], who reported new intermediate hosts in Sweden based on molecular confirmation of *T. lynciscapreoli* metacestodes in semi-domesticated reindeer. Overall, the Romanian findings are consistent with this European biogeographic pattern, suggesting that the species is not confined to isolated subpopulations but rather constitutes a stable component of the parasite fauna of the Eurasian lynx.

Although the present study is based on a single host specimen, the molecular identification of *T. lynciscapreoli* in the Eurasian lynx is epidemiologically relevant for wildlife in Romania and contributes to a better understanding of trophic relationships between the predator (Eurasian lynx) and ungulate populations within Carpathian ecosystems. Furthermore, in the current national context—where available studies have focused mainly on zoonotic helminths in wild carnivores in general or on cardiopulmonary nematodes and filarioid infections in wild felids [8,9,10]—important knowledge gaps still remain regarding the parasite fauna of Eurasian lynx in Romania. In this context, the genetic confirmation of *T. lynciscapreoli* in the Eurasian lynx contributes to the mapping of this cestode’s European distribution.

To our knowledge, this case report represents the most southern identification of *T. lynciscapreoli* within the broader distribution of Eurasian lynx. Nevertheless, the small sample size and the inability to estimate true prevalence at regional or national level represent its main limitations. Future work should include investigations of metacestodes in intermediate hosts (particularly roe deer) as well as systematic post-mortem examinations of Eurasian lynx specimens, especially opportunistically recovered road-killed individuals. Such an approach would improve knowledge on the distribution, diversity, and parasite burden of *T. lynciscapreoli* in Romania, and could also help clarify its transmission patterns within Carpathian ecosystems.

## 5. Conclusions

This study reports, for the first time, the molecular identification of *Taenia lynciscapreoli* in the Eurasian lynx from Romania and, to our knowledge, constitutes the first record from Southeastern Europe, demonstrating the presence of the parasite in the Carpathian region based on sequencing of the mitochondrial markers *cox1*, *12S rRNA*, and *nad1*.

Phylogenetic inference based on the *cox1* fragment placed the Romanian isolate within the European lineage of the species, distinct from the Chinese clade, supporting a European biogeographic pattern and suggesting parasite circulation consistent with the distribution of the definitive host and intermediate hosts (particularly the roe deer) in Carpathian habitats. Future studies should also investigate the potential health impact of such infections and include host-related data such as age, sex, and nutritional status, in order to improve the interpretation of parasite findings in a protected wildlife species.

Further studies are needed to expand molecular surveillance of definitive hosts (Eurasian lynx) and intermediate hosts (particularly roe deer and other potential cervids) in order to clarify the ecology, host specificity, and regional dynamics of this cestode. Beyond their parasitological significance, such data may also contribute to wildlife health assessment and support conservation and management strategies for the Eurasian lynx as a protected species.

## Figures and Tables

**Table 1:** Oligonucleotide primers used for PCR amplification of the mitochondrial gene fragments analyzed in this study.

Gene Marker	Primer Name	Sequence (5′–3′)	Reference
*cox1*	EgCOI1	TTTTTTGGCCATCCTGAGGTTTAT	Bowles et al., 1992 [19]; Bart et al., 2006 [20]
	EgCOI2	TAACGACATAACATAATGAAAATG	Bowles et al., 1992 [19]; Bart et al., 2006 [20]
*12S rRNA*	P60	TTAAGATATATGTGGTACAGGATTAGATACCC	Dinkel et al., 2004 [21]
	P375	AACCGAGGGTGACGGGCGGTGTGTACC	Dinkel et al., 2004 [21]
*nad1*	JB11	AGATTCGTAAGGGGCCTAATA	Bowles and McManus, 1993 [22]
	JB12	ACCACTAACTAATTCACTTTC	Bowles and McManus, 1993 [22]

## Data Availability

The original contributions presented in this study are included in the article. Further inquiries can be directed to the corresponding author(s).
